# Hypoalbuminemia predicts inferior outcome in patients with AIDS-related lymphoma

**DOI:** 10.1186/s13027-022-00448-w

**Published:** 2022-06-18

**Authors:** Jinxin Zhang, Zhiman Xie, Shaohang Cai, Shanfang Qin, Guangjing Ruan, Aili Lu, Yihua Wu, Juanjuan Chen, Jie Peng

**Affiliations:** 1 Department of Infectious Diseases, Nanfang Hospital, Southern Medical University, No. 1838 Guangzhou Avenue, Guangzhou, China; 2 Department of Infectious Diseases, the Fourth Hospital of Nanning, Nanning, China; 3 Guangxi AIDS Diagnosis and Treatment Quality Control Center, Longtan Hospital of Guangxi Zhuang Autonomous Region, Liuzhou, China; 4 Department of Respiratory Medicine, University of Chinese Academy of Sciences Shenzhen Hospital, Guangzhou, China

**Keywords:** AIDS-related lymphoma, Serum albumin, Human immunodeficiency virus, Prognostic factor, Overall survival

## Abstract

**Background:**

The prognostic value of serum albumin in acquired immunodeficiency syndrome (AIDS)-related lymphoma (ARL) remains covered.

**Methods:**

We retrospectively analyzed de novo ARL patients from 2013 to 2019 across three centers. Factors correlated with progression-free survival (PFS) and overall survival (OS) were evaluated in Kaplan–Meier, univariate and multivariate Cox proportional hazard models.

**Results:**

A total of 86 ARL patients were enrolled with a median follow-up of 34 months. In the cohort, the OS and 2-year PFS rates were 37.5% and 35.4%, respectively. In multivariate models, older age (PFS, hazard ratios [HR] = 1.035, *p* = 0.037; OS, HR = 1.034, *p* = 0.041) and hypoalbuminemia (OS, HR = 0.910, *p* = 0.038) predicted inferior survival. ARL patients with hypoalbuminemia showed worse OS and 2-year PFS (*p* = 0.028 and *p* = 0.01, respectively), which was associated with poor Eastern Cooperative Oncology Group performance status (ECOG PS) and higher International Prognosis Index (IPI) score.

**Conclusion:**

In conclusion, serum albumin at diagnosis is an independent prognostic factor for overall survival in AIDS-related lymphoma.

## Background

The introduction of combined antiretroviral therapy (cART) has markedly improved the outcome of people living with human immunodeficiency virus (PLWHIV) [1]. Nevertheless, acquired immunodeficiency syndrome (AIDS) -related lymphoma (ARL) remains a leading cause of malignancies morbidity and mortality for PLWHIV, even in the immunochemotherapy era [2–5]. Approximately 70%-90% of ARL is high-grade B-cell lymphoma, such as diffuse large B cell lymphoma (DLBCL) and Burkitt’s lymphoma (BL) [6–8]. Great heterogeneity in survival exists for DLBCL and BL patients [9]. The International Prognostic Index (IPI) incorporating simple clinical parameters remains widely used today. However, its prognostic significance was impaired for subgroup with long-term survival clearly < 50%, especially in PLWHIV [9].

Recently, several biomarkers have been suggested to be related to the prognosis of ARL, including IPI, age-adjusted IPI, CD4 + T cells count, age, Eastern Cooperative Oncology Group performance status (ECOG PS), chemotherapy, lactate dehydrogenase (LDH), Epstein- barr virus deoxyribonucleic acid (EBV DNA), hepatitis C virus, the Burkitt/Burkitt-like lymphoma subtype, history of clinical AIDS, and expression of B-cell lymphoma-2 (BCL2), cluster of differentiation 4 (CD44), Protein 53 (p53) and Immunoglobulin M (IgM) [10–15]. Alternatively, recent immunosuppression and prolonged HIV viraemia have important independent roles in the development of ARL [16]. Otherwise, unclassifiable histology, stage III or IV, and no concomitant cART during chemotherapy were independently associated with a higher relapse rate [17].

Nevertheless, it is important to find the simple prognostic marker to identify ARL patients with different outcomes, especially in low-income and middle-income countries with poor infrastructure for cancer management. We therefore performed this study to evaluate the risk factors in ARL patients and would provide useful prognostic information for clinicians.

## Methods

### Ethical considerations

The study was performed in accordance with the Declaration of Helsinki and was approved by the Institutional Ethics Committee of Nanfang Hospital (study identifier NFEC-2021–178). The committee decided to waive the need for written informed consent from the participants in the study because the data were analyzed retrospectively and anonymously.

### Patients and data collection

This retrospective multicenter study was conducted at Nanfang Hospital Affiliated with Southern Medical University, Fourth Hospital of Nanning, and Longtan Hospital of Guangxi Zhuang Autonomous Region. A total of 143 hospitalized de novo HIV-positive lymphoma patients from 2013 to 2019 were included. 86 patients with ARL, whose survival state were definite, were enrolled in the present study. The deadline for the follow-up was 2021. The pathological diagnosis of lymphoma was based on the 2008 World Health Organization (WHO) classification [18].

The clinical variables were evaluated, including date of enrollment, history of HIV/AIDS, histological subtype, sex, age, body mass index (BMI), baseline CD4 + T cells count, cluster of differentiation 8 (CD8 +) T cells count, CD4/CD8 ratio, erythrocyte sedimentation rate (ESR), complete blood cell count (CBC), LDH, serum albumin (ALB), red cell distribution width (RDW) ratio, Lugano classification, ECOG PS, and IPI score. All data were expressed as means ± standard deviation (SD) or median or percentage when appropriated.

### Clinical assessments

Computed tomography (CT) or 18F-fluorodeoxyglucose positron emission tomography/computed tomography (FDG PET/CT) was performed for radiological evaluation. Brain magnetic resonance imaging (MRI) was used to assess central nervous system involvement. Overall survival (OS) was defined as the time from diagnosis of ARL to the last follow-up or death from any cause. The 2-year progression-free survival (PFS) was defined as the time from diagnosis of ARL until progression, relapse, or death from any cause at 24 months.

### Statistical analysis

Statistical analysis was performed by the Statistical Package for the Social Sciences (SPSS) version 17. OS and 2-year PFS were estimated by the Kaplan–Meier method and compared by the log-rank test. Distributions of clinical characteristics between the different groups were carried out by students’ test or Fisher’s exact test. Linear correlation analysis and one way analysis of variance (ANOVA) were used to analyze the relationship of ALB with other factors. The univariate and multivariate analysis was performed by Cox proportional hazard model. Hazard ratios (HR) and 95% confidence interval (CI) were used to summarize the association between variables and survival. Bivariate correlations among variables were carried out using Pearson's correlation test. All *p* values were two-sided, and the significance was defined as *p* < 0.05.

## Results

### Demographic and clinical characteristics in ARL patients enrolled

A total of 86 patients with ARL were enrolled, with a median follow-up of 34 months. DLBCL is the most frequent subtype in ARL (95.3%). There were 54 (62.8%) ARL patients dead at the end of follow-up, and these patients tended to present with higher IPI score (*p* = 0.027) and lower CD4 + T cells (*p* = 0.046), as shown in Table 1. There were no significant differences in the clinical features of sex, BMI, CD8 + T cells, CD4/CD8 ratio, ESR, platelet count, LDH, serum ALB, and ECOG PS.

**Table 1:** The demographics and clinical characteristics of patients enrolled

Characteristics	Total	Patients with AIDS-related lymphoma		*P* value
		Survival	Non-survival	
Sample size, n	86	32	54	–
Sex (male), n (%)	69 (80.2)	26 (81.3)	43 (79.6)	0.855
Age (years)	50.97 ± 13.80	47.62 ± 13.85	52.94 ± 13.52	0.084
BMI (Kg/m^2^)	21.77 ± 3.65	21.24 ± 3.51	22.12 ± 3.73	0.298
CD4 + T cells	196.73 ± 148.82	237.59 ± 159.98	170.58 ± 136.50	0.046
CD8 + T cells	617.47 ± 352.93	654.03 ± 361.04	595.53 ± 349.81	0.476
CD4/CD8 ratio	0.35 ± 0.27	0.40 ± 0.29	0.32 ± 0.26	0.184
ESR	51.08 ± 38.81	44.19 ± 41.04	55.67 ± 37.07	0.246
Platelet count	244.17 ± 113.61	234.86 ± 105.57	249.80 ± 118.83	0.560
LDH	694.21 ± 816.42	534.49 ± 704.25	792.50 ± 870.30	0.161
ALB, g/L	34.29 ± 7.49	36.01 ± 6.56	33.28 ± 7.86	0.107
RDW-SD	45.50 ± 7.91	43.55 ± 4.64	46.64 ± 9.17	0.089
*ECOG PS*				0.205
0	9 (10.5)	4 (12.5)	5 (9.3)	
1–2	64 (74.4)	26 (81.3)	38 (70.4)	
3–4	13 (15.1)	2 (6.3)	11 (20.4)	
*IPI score*				0.027
0–1	23 (26.7)	14 (43.8)	9 (16.7)	
2–3	41 (47.7)	11 (34.4)	30 (55.6)	
4–5	19 (22.1)	7 (21.9)	12 (22.2)	
Date missing	3 (3.5)	0 (0)	3 (5.6)	
*Subtypes*				
DLBCL	82 (95.3)	29 (90.6)	53 (98.1)	
BL	2 (2.3)	1 (3.1)	1 (1.9)	
Other ARL	2 (2.3)	2 (6.2)	0 (0)	

Abbreviation: AIDS, acquired immunodeficiency syndrome; BMI, body max index; CD4, cluster of differentiation 4; CD8, cluster of differentiation 8; ESR, erythrocyte sedimentation rate; LDH, lactate dehydrogenase; ALB, albumin; RDW-SD, red cell distribution width standard deviation; ECOG PS, Eastern Cooperative Oncology Group performance status; IPI, International Prognosis Index

### Prognostic factors associated with patients with ARL

**Fig. 1:**
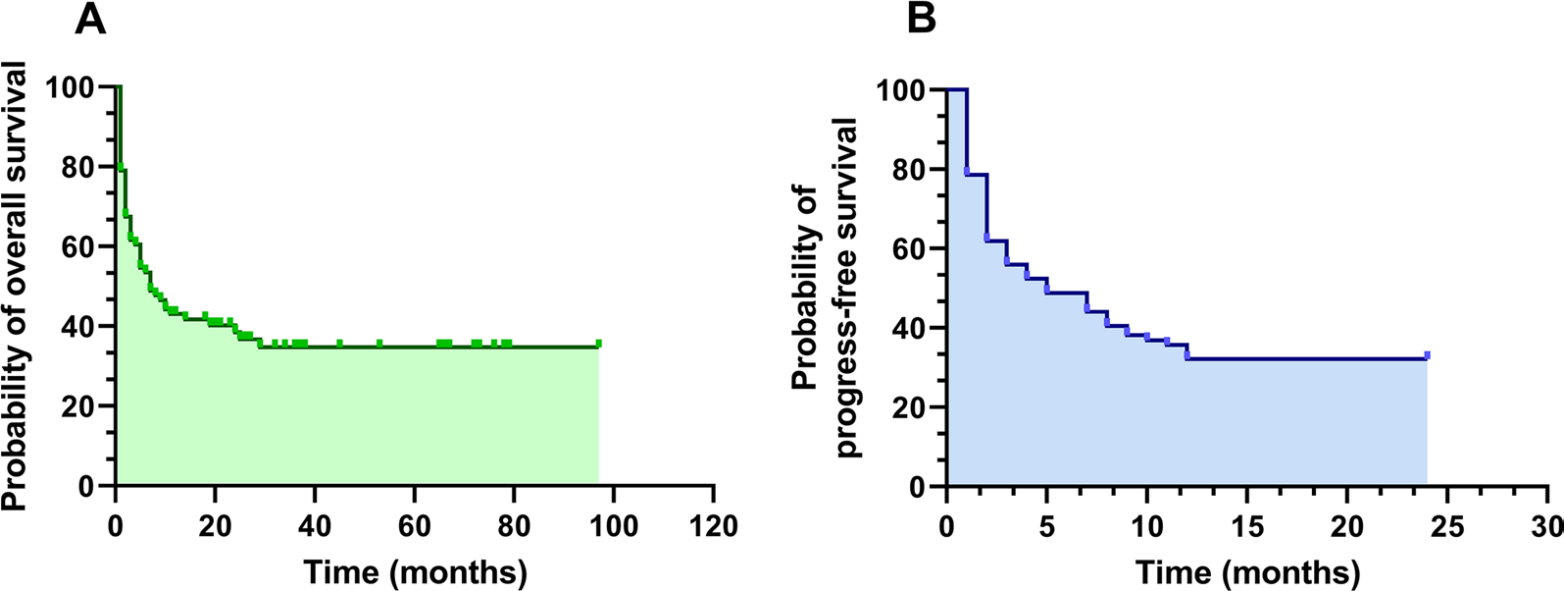
Kaplan–Meier survival curves for **A** overall survival (37.5%) and **B** 2-year progression-free survival (35.4%) in 86 AIDS-related lymphoma patients. Abbreviation: AIDS: Acquired immunodeficiency syndrome

Among ARL patients enrolled, the OS and 2-year PFS were 37.5% and 35.4%, respectively (Fig. 1). To further evaluate risk factors correlated with prognosis, univariable and multivariable analysis were conducted. Results from multivariate analysis demonstrated that older age (HR = 1.034, *p* = 0.041) and lower ALB level (HR = 0.910, *p* = 0.038) were independent prognostic factors associated with OS in ARL population (Table 2).

**Table 2:** Factors associated with overall survival in AIDS-related lymphoma patients

Variables	Univariate analysis			Multivariate analysis		
	HR	95%CI	*P*	HR	95%CI	*P*
Sex (male), n (%)	1.014	0.523–1.967	0.967	0.948	0.542–3.542	0.937
Age (years)	1.018	0.998–1.039	0.084	1.034	1.001–1.069	0.041
BMI, Kg/m^2^	1.029	0.955–1.108	0.452	1.046	0.940–1.164	0.407
CD4 + T cells	0.998	0.996–1.000	0.093	0.997	0.990–1.003	0.349
CD8 + T cells	1.000	0.999–1.001	0.756	1.001	0.999–1.003	0.416
CD4/CD8 ratio	0.451	0.412–1.438	0.178	1.697	0.034–84.374	0.791
ESR	1.004	0.997–1.012	0.234	0.998	0.987–1.009	0.683
Platelet count	1.000	0.998–1.002	0.958	1.000	0.996–1.003	0.857
LDH, U/L	1.000	1.000–1.001	0.187	1.000	1.000–1.001	0.523
ALB, g/L	0.965	0.929–1.002	0.063	0.910	0.832–0.995	0.038
RDW-SD	1.040	1.007–1.074	0.016	1.094	0.993–1.204	0.068
ECOG PS	1.255	0.957–1.644	0.100	0.708	0.396–1.266	0.244
IPI score	1.152	0.941–1.411	0.170	0.884	0.574–1.360	0.574

Abbreviation: AIDS, acquired immunodeficiency syndrome; BMI, body max index; CD4, cluster of differentiation 4; CD8, cluster of differentiation 8; ESR, erythrocyte sedimentation rate; LDH, lactate dehydrogenase; ALB, albumin; RDW-SD, red cell distribution width standard deviation; ECOG PS, Eastern Cooperative Oncology Group performance status; IPI, International Prognosis Index

We then adopted multivariable analysis to evaluate factors correlated with 2-year PFS in ARL patients enrolled. Of note, as an unfavorable factor, age (HR = 1.035, *p* = 0.041) retained its statistical significance for 2-year PFS in patients with ARL (Table 3).

**Table 3:** Factors associated with 2-year progress-free survival in patients with AIDS-related lymphoma

Variables	Univarite analysis			Multivariate analysis		
	HR	95%CI	*P*	HR	95%CI	*P*
Sex (male), n (%)	1.054	0.546–2.035	0.876	1.116	0.332–3.749	0.859
Age (years)	1.016	0.996–1.037	0.112	1.035	1.002–1.070	0.037
BMI, Kg/m^2^	1.013	0.943–1.088	0.728	0.986	0.885–1.099	0.801
CD4 + T cells	0.998	0.997–1.000	0.139	0.997	0.991–1.004	0.411
CD8 + T cells	0.962	0.999–1.001	0.962	1.001	0.999–1.004	0.237
CD4/CD8 ratio	0.497	0.161–1.533	0.224	2.246	0.068–73.659	0.650
ESR	1.005	0.998–1.012	0.199	1.002	0.991–1.013	0.777
Platelet count	1.000	0.998–1.002	0.840	0.999	0.995–1.003	0.587
LDH, U/L	1.000	1.000–1.001	0.183	1.000	1.000–1.001	0.499
ALB, g/L	0.958	0.924–0.994	0.023	0.945	0.867–1.031	0.206
RDW-SD	1.035	1.002–1.068	0.037	1.063	0.970–1.166	0.193
ECOG PS	1.342	1.039–1.732	0.024	0.913	0.522–1.598	0.751
IPI score	1.216	1.000–1.480	0.050	1.003	0.662–1.519	0.990

Abbreviation: AIDS, acquired immunodeficiency syndrome; BMI, body max index; CD4, cluster of differentiation 4; CD8, cluster of differentiation 8; ESR, erythrocyte sedimentation rate; LDH, lactate dehydrogenase; ALB, albumin; RDW-SD, red cell distribution width standard deviation; ECOG PS, Eastern Cooperative Oncology Group performance status; IPI, International Prognosis Index

### Hypoalbuminemia predicted worse prognosis in ARL

**Fig. 2:**
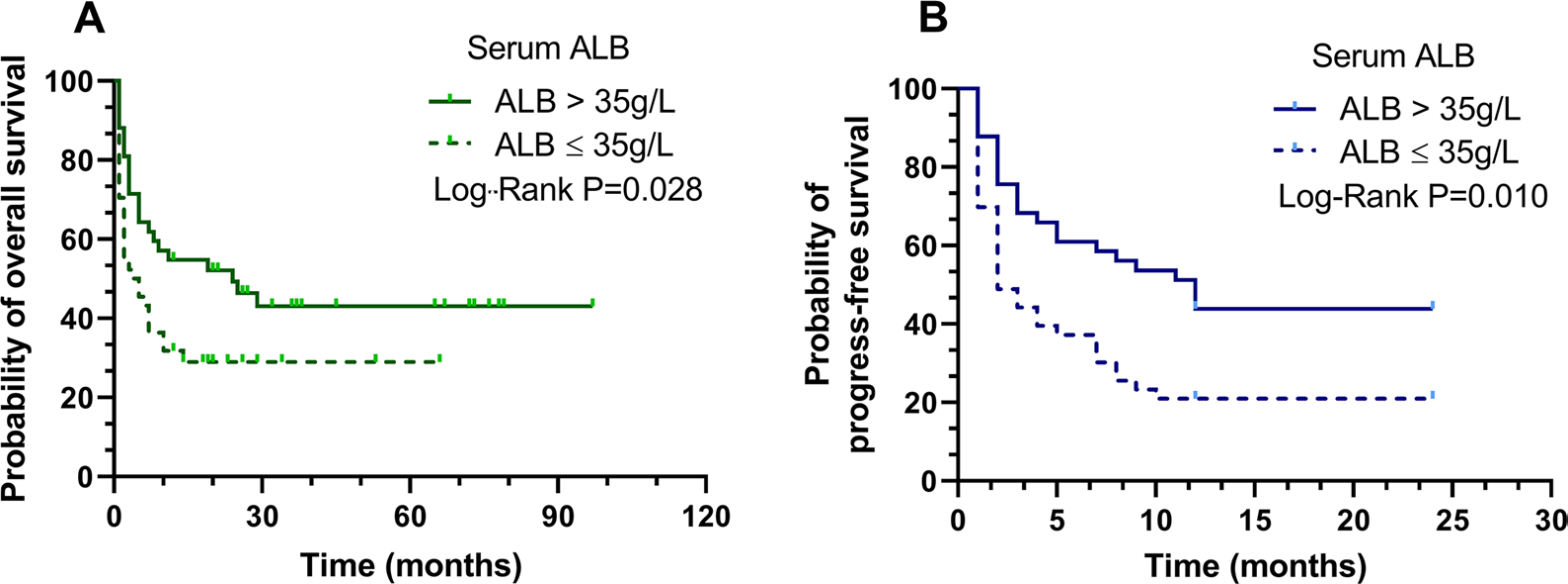
Kaplan–Meier survival curves of the serum ALB stratifications for overall survival (**A**) and 2-year progression-free survival (**B**) in AIDS-related lymphoma patients (n = 86). Abbreviation: ALB, albumin; AIDS: Acquired immunodeficiency syndrome

To further investigate ALB as a novel biomarker for prognosis in ARL, we divided patients into ALB > 35.0 g/L and ALB ≤ 35.0 g/L groups at the time of diagnosis. A Kaplan–Meier survival analysis was conducted by using data from all patients enrolled in the study. Similarly, the patients with low ALB level had remarkably worse outcomes in terms of OS (*p* = 0.028) and 2-year PFS (*p* = 0.010), when compared with those with high ALB level, as depicted in Fig. 2.

### Relationship between serum album level and clinical variables

**Fig. 3:**
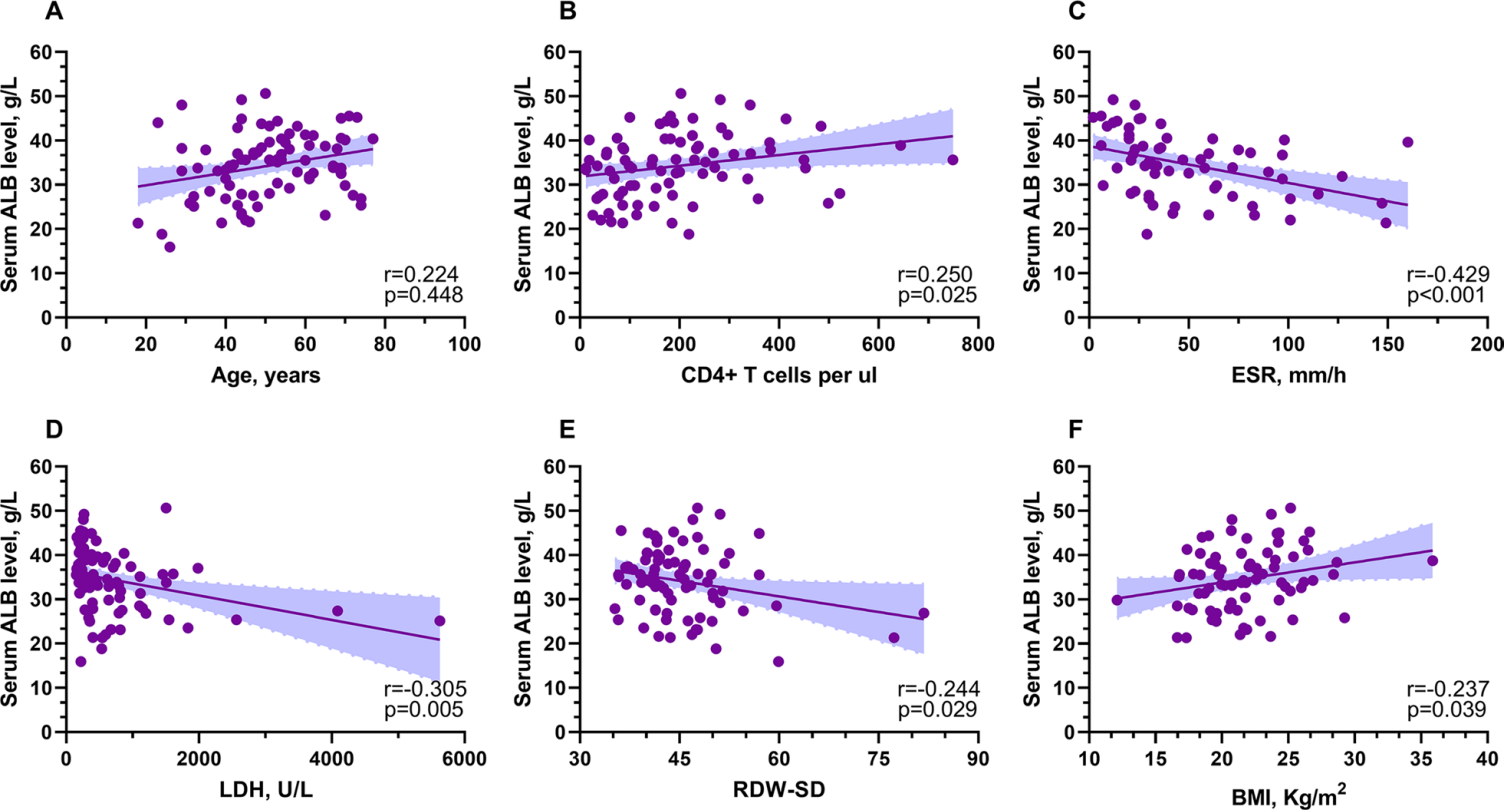
Correlation of serum ALB with other clinical variables. ALB level by age (**A**), CD4 + T cells (**B**), ESR (**C**), LDH (**D**), RDW-SD (**E**), and BMI (**F**). Abbreviations: ALB, albumin; CD4: Cluster of differentiation 4; ESR, erythrocyte sedimentation rate; LDH, lactate dehydrogenase; RDW-SD, red cell distribution width standard deviation; BMI, body max index

We next explored the relationship between serum album level and other clinical variables in patients with ARL by Pearson's correlation analysis. A strong correlation between serum ALB and age (r = 0.263, *p* = 0.016), CD4 + T cells (r = 0.250, *p* = 0.025), ESR (r = -0.429, *p* < 0.001), LDH (r = -0.305, *p* = 0.005), RDW-SD (red cell distribution width standard deviation, r = -0.244, *p* = 0.029), and BMI (r = 0.237, *p* = 0.039) were validated (Fig. 3).

**Fig. 4:**
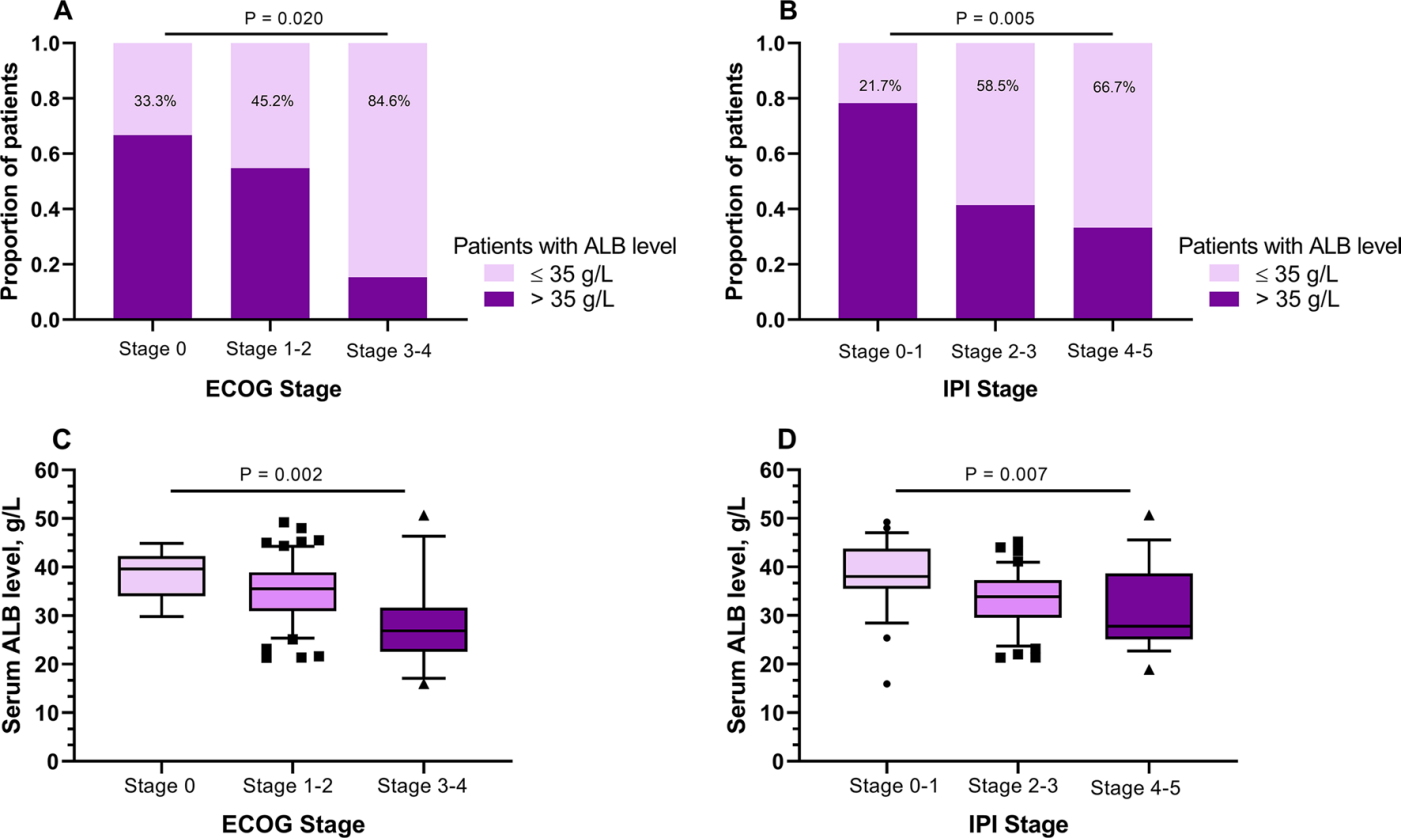
Association between serum ALB and ECOG PS (**A**) as well as IPI score (**B**). The average ALB level in ECOG PS groups (**C**) and IPI score groups (**D**). Abbreviations: ALB, albumin; IPI, International Prognosis Index; ECOG PS, Eastern Cooperative Oncology Group performance status

A total of 33.3%, 45.2%, and 84.6% patients had low ALB level in ECOG PS 0, 1–2, and 3–4 groups (*p* = 0.002). Similarity, in patients with IPI score 0–1, 2–3, and 4–5, a total of 21.75%, 58.5% and 66.7% population, respectively, had low ALB level (*p* = 0.005). We therefore analyzed the ALB level in each ECOG PS group and IPI score group. The average serum ALB level was 38.47 ± 4.89, 34.98 ± 6.74, and 28.08 ± 9.17 g/L in ECOG PS 0, 1–2 and 3–4 groups respectively (*P* = 0.002). A similar tendency was observed in terms of IPI score groups with 38.27 ± 7.39, 33.36 ± 5.98, and 31.61 ± 8.67 g/L (*p* = 0.007), as shown in Fig. 4.

## Discussion

Patients with hypoalbuminemia (≤ 35.0 g/L) are considered to have malnutrition. Malnutrition is common in HIV infection and plays an independent and significant role in its morbidity and mortality [19, 20]. Low serum albumin at diagnosis has been identified as a simple prognostic factor in some cancers including non-Hodgkin's lymphoma (NHL) [21–25] which comprises more than 50% of all AIDS-defining cancers. However, its role in predicting clinical outcomes of AIDS-related lymphoma has not been evaluated.

In this study, we retrospectively confirmed that hypoalbuminemia was not uncommon at diagnosis of de novo ARL and was associated with poor survival outcome attractively. A similar trend was observed in the analyses of HIV-negative DLBCL patients [22, 24]. We also uncovered a strong relationship between hypoalbuminemia and poor ECOG PS as well as higher IPI score. As reported, IPI score and ECOG PS at diagnosis had been identified as independent risk factors for survival in ARL population and in HIV-negative lymphoma patients [9, 15, 26, 27]. These associations with adverse prognostic factors translated into shorter OS for ARL patients with hypoalbuminemia. This indicated that serum albumin level may be not only a surrogate of poor nutritional, but also driven by the aggressive tumor behavior and inflammatory status [24, 28], which was correlated with shorter survival, treatment response, treatment-related toxicity and compliance [22].

In the present study, we demonstrated that age was another important independent predictor of OS and 2-year PFS in patients with ARL. Our data resembles the result of a Swedish study which finds that age is the most important predictor of survival in HIV-negative DLBCL patients [29]. In fact, a study of 100 HIV-positive lymphoma patients proved that age may be a significant prognostic factor for 2-year OS [10]. Hence, age is generally associated with adverse prognosis in ARL. We furthermore confirmed that ALB is another important risk factor in patients with ARL.

The strengths of the study include the multicenter, population-based design, the long duration of follow-up and the relatively large number of patients with well-documented data. The main limitation of this study is its retrospective nature, which may have caused a selection bias. To overcome these problems, we applied multivariable analysis to adjust for confounding factors. Another caution is that internal and external validation is needed before applying the serum ALB to clinical practice. These factors should be clarified in future prospective studies.

## Conclusion

In summary, we showed that hypoalbuminemia is a simple and effective prognostic factor in AIDS-related lymphoma patients. For ARL patients with hypoalbuminemia, closer follow-up and timely intervention are necessary. Further research is needed to confirm whether albumin supplementation is required for those subpopulations.

## Data Availability

The data are presented in the manuscript.

## Abbreviations

AIDS: Acquired immunodeficiency syndrome
ALB: Albumin
ANOVA: Analysis of variance
ARL: Acquired immunodeficiency syndrome-related lymphoma
BCL-2: B-cell lymphoma-2
BL: Burkitt’s lymphoma
BMI: Body mass index
cART: Combined antiretroviral therapy
CBC: Complete blood cell count
CD4: Cluster of differentiation 4
CD8: Cluster of differentiation 8
CI: Confidence interval
CT: Computed tomography
DLBCL: Diffuse large B cell lymphoma
EBV DNA: Epstein–Barr virus deoxyribonucleic acid
ECOG PS: Eastern Cooperative Oncology Group performance status
ESR: Erythrocyte sedimentation rate
FDG PET: F-fluorodeoxyglucose positron emission tomography
HIV: Human immunodeficiency virus
HR: Hazard ratios
IgM: Immunoglobulin M
IPI: International Prognosis Index
LDH: Lactate dehydrogenase
MRI: Magnetic resonance imaging
NHL: Non-Hodgkin’s lymphoma
OS: Overall survival
P53: Protein 53
PFS: Progression-free survival
PLWHIV: People living with human immunodeficiency virus
RDW: Red cell distribution width
SD: Standard deviation
SPSS: Statistical Package for the Social Sciences
WHO: World Health Organization
